# Mechanisms and disease associations of oxidative stress-mediated brain-bone axis dysregulation: a knowledge mapping and trend analysis based on Bibliometrics

**DOI:** 10.3389/fnagi.2026.1755503

**Published:** 2026-06-15

**Authors:** Hao Zeng, Zhengpeng Li, Siyuan Jiang, Jinjie Wu, Zijing Ren, Yahui Leng, Qiang Zhao, Peiyang Zhou, Ping Gao, Puqing Wang, Xuanxuan Zou

**Affiliations:** 1 Hubei Provincial Clinical Research Center for Parkinson’s Disease, Xiangyang Key Laboratory of Movement Disorders, Xiangyang No.1 People's Hospital, Hubei University of Medicine, Xiangyang, China; 2 College of Zhuang Medicine, Guangxi University of Chinese Medicine, Nanning, China; 3 HIM-BGI Omics Center, Hangzhou Institute of Medicine (HIM), Chinese Academy of Sciences, Zhejiang, China; 4 Faculty of Health Sciences, University of Macau, Macau, China; 5 Department of Neurology, Xiangyang No.1 People’s Hospital, Hubei University of Medicine, Xiangyang, China; 6 Department of Radiology, Hubei Provincial Clinical Research Center for Parkinson’s Disease, Xiangyang No.1 People's Hospital, Hubei University of Medicine, Xiangyang, China

**Keywords:** Bibliometrics, brain-bone axis, knowledge mapping, neuro-skeletal crosstalk, oxidative stress

## Abstract

**Background:**

Oxidative stress, characterized by the systemic imbalance between reactive oxygen species and antioxidant defenses, is increasingly recognized as a central pathological nexus driving the dysregulation of the brain-bone axis. Despite the accumulation of empirical evidence, a systematic characterization of the field’s intellectual structure and thematic progression remains absent. This study employs a multi-database bibliometric approach to map the research landscape of oxidative stress-mediated neuro-skeletal crosstalk and identify emerging research frontiers.

**Methods:**

A systematic search was performed across the Web of Science Core Collection, Scopus, and PubMed databases from their inception to April 30, 2025. Following a rigorous screening process based on predefined criteria, 717 relevant publications were included. Bibliometric mapping and network analyses were conducted using CiteSpace, VOSviewer, and the Bibliometrix R-package to evaluate collaboration patterns, co-citation structures, and keyword evolution.

**Results:**

The analysis reveals a steady increase in research output since 2009, marked by distinct developmental phases. Early investigations primarily focused on fundamental oxidative damage mechanisms, while subsequent research transitioned toward systemic disease associations (e.g., Alzheimer’s disease and osteoporosis) and targeted intervention strategies, including mesenchymal stem cell therapy and melatonin. Recent trends indicate a methodological and thematic shift toward high-resolution translational themes, such as neuroimmune regulation and the “gut-brain-bone” axis. Notably, “gut microbiota” and “extracellular vesicles” have emerged as high-centrality nodes, reflecting an increasing focus on inter-organ communication and systemic redox modulation.

**Conclusion:**

This study provides the first comprehensive mapping of the research trajectories within the oxidative stress-mediated brain-bone axis field. The findings delineate a clear progression from isolated mechanistic studies toward integrated, multi-system frameworks, underscoring the shift toward precision medicine and translational applications. By identifying current research gaps and emerging hotspots, this analysis offers a systematic reference for future interdisciplinary investigations and the development of targeted therapeutic strategies for neuro-skeletal comorbidities.

## 1 Introduction

Oxidative stress, characterized by a systemic imbalance between reactive oxygen species (ROS) and antioxidant defense systems, serves as a critical molecular nexus connecting the nervous and skeletal systems (Guo et al., 2023; Liu et al., 2025). In neurodegenerative conditions such as Alzheimer’s disease and Parkinson’s disease, ROS accumulation drives neuronal apoptosis and synaptic impairment by inducing mitochondrial dysfunction and neuroinflammatory cascades (Bai et al., 2022; Wen et al., 2025). Concurrently, excessive ROS within the skeletal microenvironment inhibits osteoblast differentiation while accelerating osteoclast-mediated bone resorption, contributing to metabolic disorders such as osteoporosis and osteoarthritis (Amirkhizi et al., 2023; Zhang et al., 2023a,b). The emerging “Brain-Bone Axis” theory has characterized a sophisticated bidirectional regulatory network between these systems. The central nervous system modulates bone remodeling through neuroendocrine pathways and sympathetic activity (Shi and Chen, 2024; Zhang and Zhang, 2024), while bone-derived factors, such as osteocalcin and fibroblast growth factors (FGF), cross the blood-brain barrier to influence neurogenesis and cognitive function (Gao et al., 2025). Crucially, oxidative stress is increasingly viewed not merely as a localized driver of damage but as a “common soil” mechanism that mediates the coordinated dysregulation of the brain-bone axis via signaling pathways such as NF-κB, Nrf2, and mTOR (Cordaro et al., 2021; Liu et al., 2024; Perluigi et al., 2021).

Understanding this cross-organ vicious cycle could provide a novel framework for addressing neuro-skeletal comorbidities. However, current literature presents two primary limitations. First, research remains largely fragmented, with a predominant focus on single organs or specific diseases, resulting in a lack of integrated evidence regarding the systemic associations within the brain-bone axis. Second, the methodological disconnection between neuroscience and bone biology has hindered the identification of cross-scale regulatory patterns. To address these challenges, there is an urgent need for a systematic, quantitative examination of the research landscape to reconcile these disparate findings. Bibliometric analysis offers a rigorous, data-driven approach to mapping the intellectual structure and thematic evolution of a scientific field (Ninkov et al., 2022). Unlike traditional narrative reviews, which may be constrained by subjective interpretation, bibliometric tools—including CiteSpace, VOSviewer, and the bibliometrix R-package—enable the objective identification of knowledge foundations and emerging research trends. By systematically retrieving literature from the Web of Science Core Collection (WoSCC), Scopus, and PubMed from their inception to April 30, 2025, this study constructs a multi-dimensional knowledge map of “oxidative stress-mediated brain-bone axis dysregulation.” This analysis aims to delineate the global collaboration networks and thematic shifts within the field, providing a structured reference for understanding neuro-skeletal interactions and offering a data-driven foundation for future research strategies.

## 2 Methods

### 2.1 Literature retrieval and screening

**Figure 1:**
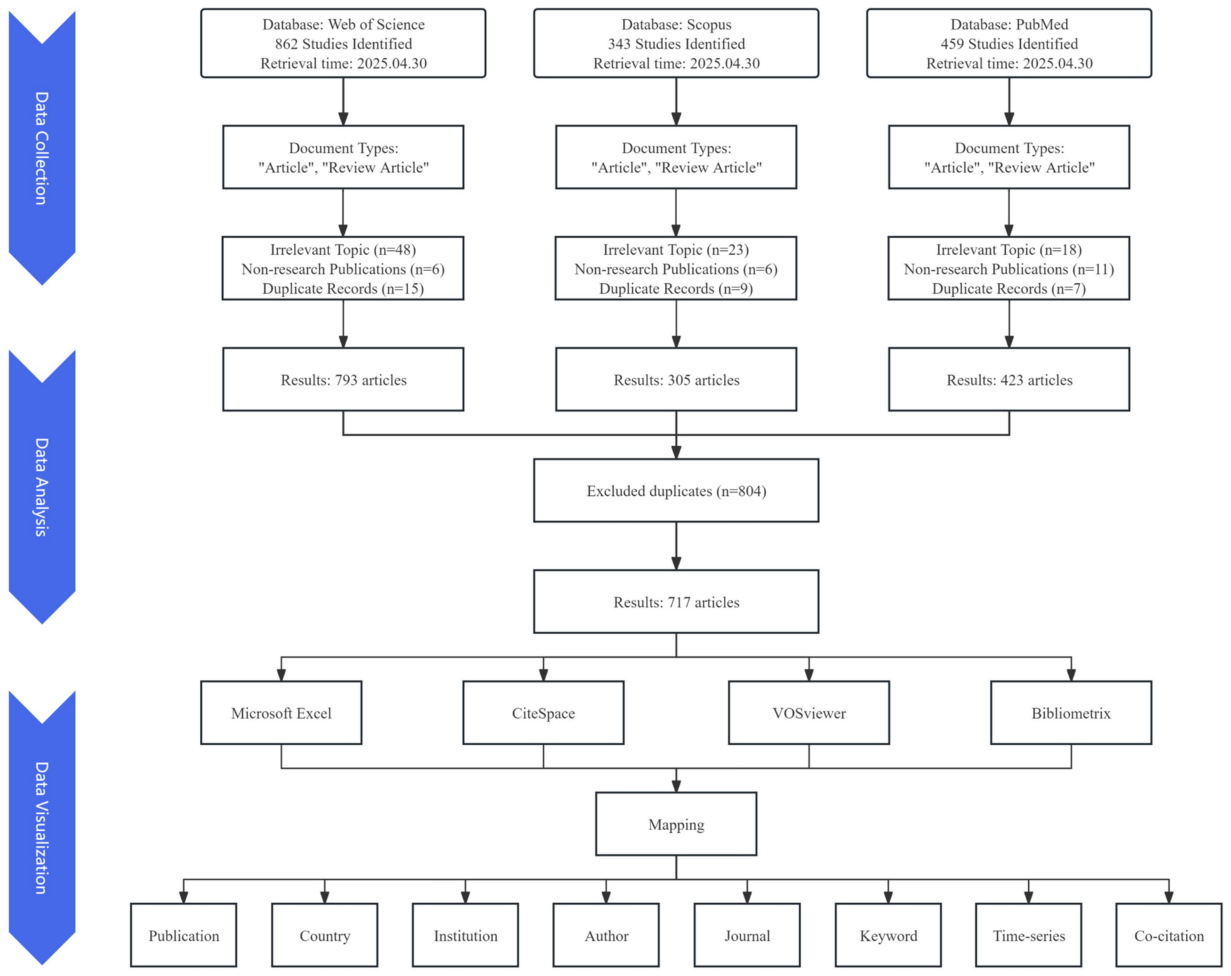
Inclusion and exclusion process for studies on oxidative stress-mediated brain-bone axis dysregulation.

The systematic literature search was conducted across three authoritative databases: WoSCC, Scopus, and PubMed. These platforms were selected for their comprehensive coverage and high-impact indexing of global biomedical literature (Kokol et al., 2021; Permana and Lukito, 2024; Yeung, 2023). As illustrated in the primary workflow (Figure 1), the retrieval encompassed all records from each database’s inception through April 30, 2025, utilizing an advanced search strategy with Boolean operators to combine terms related to “oxidative stress,” “brain,” and “bone.”

To ensure methodological rigor, the initial 1,664 identified records (862 from WoSCC, 343 from Scopus, and 459 from PubMed) underwent a structured multi-stage screening process. Document types were strictly limited to “Articles” and “Reviews.” Records were systematically excluded based on irrelevant topics (*n* = 89), non-research nature (*n* = 23), or internal duplication (*n* = 31). Following the merging of datasets and a comprehensive cross-database deduplication, 804 duplicate records were removed. A final dataset of 717 eligible publications was established for subsequent analysis (Figure 1). To quantify the inter-rater reliability, the Cohen’s kappa coefficient was assessed between two independent reviewers.

### 2.2 Data standardization and visualization analysis

The validated records were exported in standardized formats (Plain Text for WoSCC, CSV for Scopus, and TXT for PubMed) and managed via EndNote (v.21.0). Following the visualization framework (Figure 1), four analytical platforms were employed. Microsoft Excel 2024 was used for fundamental statistical syntheses, such as annual publication trends and subject distribution. VOSviewer (v.1.6.20) was utilized to construct keyword co-occurrence and collaboration networks (Kirby, 2023), employing the association strength method for network normalization. In the generated maps, node size and color represent different categories, while link thickness reflects the strength of association. The analysis thresholds were set to a minimum cluster frequency of 5 to balance network interpretability with information integrity. CiteSpace (v.6.4. R1) was applied to delineate the structural and temporal evolution of the field, including country, institution, author, and co-citation analysis. The visualization parameters were configured with a time slicing period of 2 years, and the g-index (k = 25) was set as the node selection criterion. To simplify the network and highlight key connections, the Pathfinder algorithm was applied for network pruning. Cluster labeling was automatically generated using the Log-Likelihood Ratio (LLR) algorithm. Finally, a comprehensive analysis at the macro level was performed on the bibliographic information of the aforementioned 717 screened publications using the bibliometrix R-package (version 5.0) (Aria and Cuccurullo, 2017; Susilawati et al., 2025). All analyses adhered to standardized metadata preprocessing and frequency-based inclusion thresholds, ensuring that items not meeting the criteria were systematically excluded.

## 3 Results

### 3.1 Annual trends and distribution of publications

**Figure 2:**
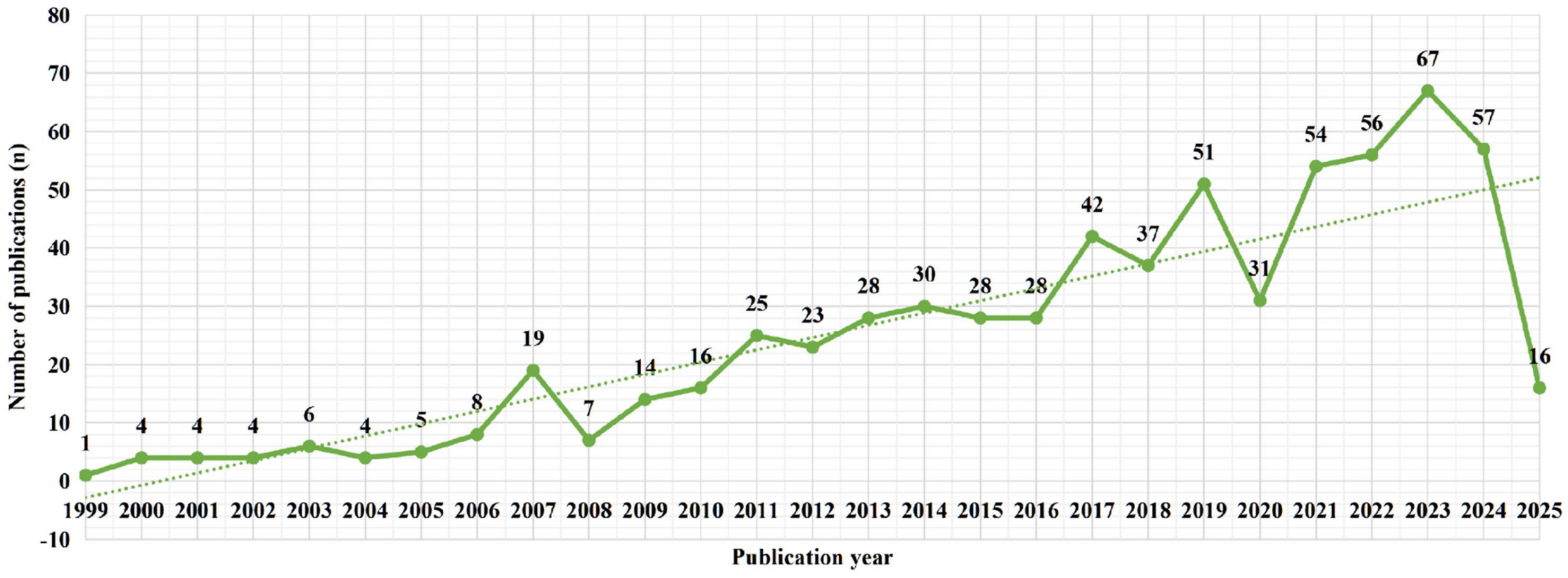
Trend chart of publications. A line chart showing the number of publications in the field of oxidative stress-mediated brain-bone axis disorders in the WoSCC database from 1999 to 2025.

Following the initial retrieval of 1,664 records, 717 articles were ultimately included for analysis after the application of inclusion/exclusion criteria and independent investigator review. Publication output in this field exhibited periodic fluctuations within an overall upward trend (Figure 2). From 1999 to 2008, the average annual number of publications remained below 10. A steady expansion was observed after 2009, with the annual output exceeding 40 for the first time in 2017. A decline to 30 publications occurred in 2020, followed by a subsequent rise in 2022–2023, reaching a peak of over 60. In 2024, the output totaled 57. As the data collection window concluded at the end of April 2025, the 16 publications identified from January to April 2025 reflect only partial annual data. Over the past decade, the field has experienced a cumulative growth exceeding 600%.

### 3.2 Global distribution and contributions of publications

**Figure 3:**
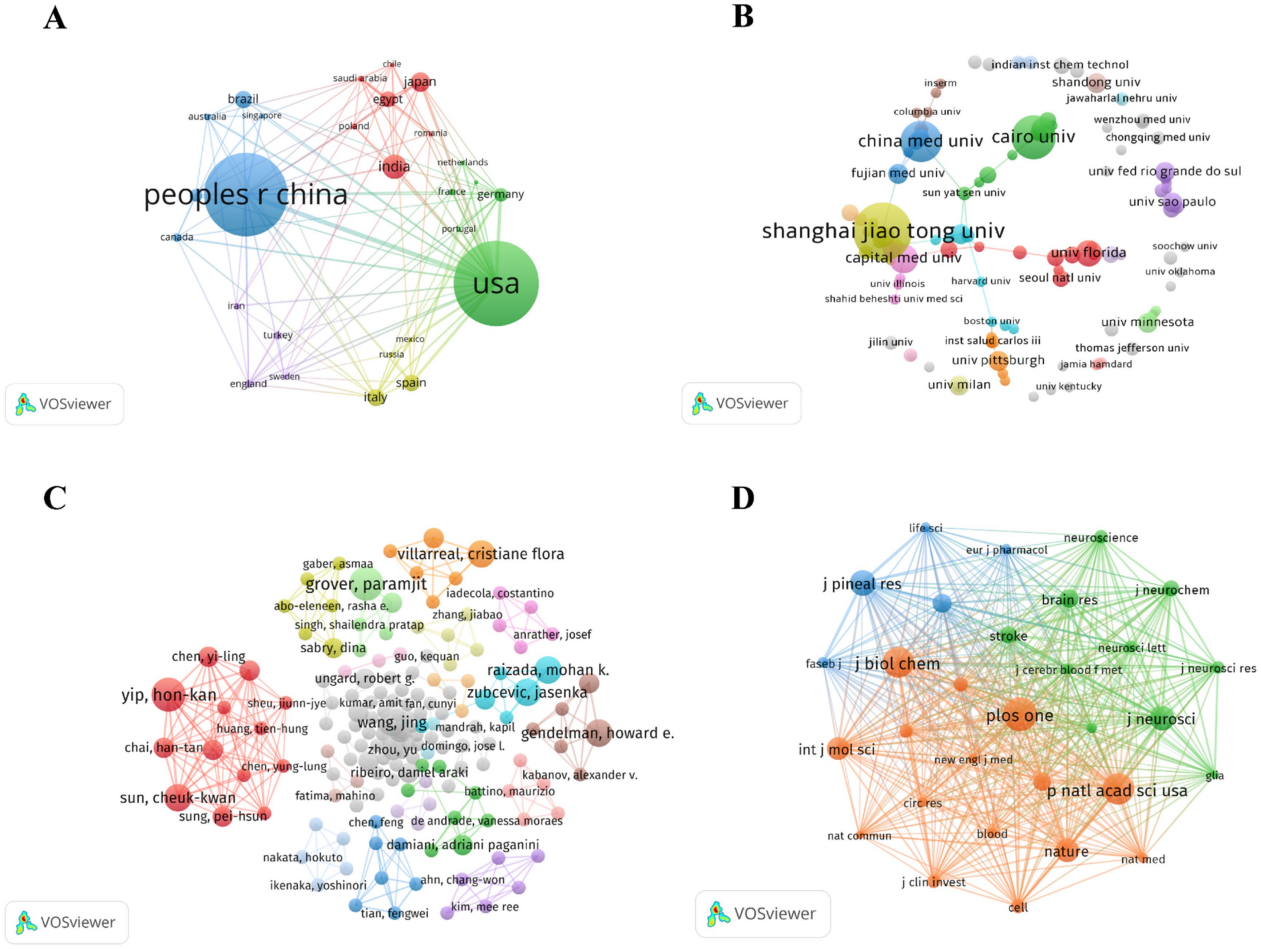
Visualized co-occurrence maps of countries/institutions/authors/journals in the research field of oxidative stress-mediated brain-bone axis dysregulation. **(A)** Country co-occurrence map, where nodes represent countries, node size corresponds to publication volume, and connecting lines indicate inter-country collaboration. **(B)** Institutional co-occurrence map, featuring nodes as institutions, with node size reflecting publication output and links denoting institutional partnerships. **(C)** Author co-occurrence map, displaying authors as nodes sized by their publication count, and connections showing collaborative relationships between authors. **(D)** Journal co-occurrence map, with journals represented as nodes proportional to their publication volume, and linkages illustrating thematic or citation-based associations between journals.

Bibliometric analysis revealed a distinct differentiation in research contributions across countries and regions. The United States leads in both productivity (172 publications) and citation impact, with a total of 11,533 citations (Table 1). China ranks second in publication volume with 170 articles, yet maintains a total citation count of 4,133 (Table 1). Notably, the United States leads in the number of publications (172), total citations (11533), and average citations per paper (67.0523) among all countries in this field, underscoring its dominant position in both research output and academic impact. Within Asia, India and Japan were the primary contributors to publication output, while Brazil was the sole representative from South America among the top-ranked nations. Among European nations, Italy and Germany exhibited high average citations per publication. Collectively, the combined output from the United States and China accounted for approximately 47.7% of the total publications. The top 10 countries/regions by publication volume are summarized in Table 1, and their collaborative network is visualized in Figure 3A.

**Table 1:** Top 10 countries in publication volume about oxidative stress-mediated brain-bone axis dysregulation research.

Rank	Country	Number of publications	Number of total citations	Number of average citations
1	United States	172	11,533	67.0523
2	China	170	4,133	24.3118
3	India	54	1,593	29.5
4	Japan	42	1,435	34.1667
5	Brazil	35	933	26.6571
6	Italy	34	1,446	42.5294
7	Egypt	32	525	16.4062
8	Spain	32	1,074	33.5625
9	South Korea	27	694	25.7037
10	Germany	26	1,344	51.6923

### 3.3 Distribution and contribution of research institutions

Analysis of contributing institutions revealed distinct patterns of productivity and institutional collaboration. Shanghai Jiao Tong University ranked first in publication volume (*n* = 19), followed by China Medical University (*n* = 13) and Capital Medical University (*n* = 9). Among the U.S. institutions, the University of Florida accounted for 745 total citations from 8 publications, while Harvard Medical School received 313 citations from 6 publications (Table 2). Institutions from Egypt, notably Cairo University and the National Research Centre, were also identified among the most productive. The institutional collaboration network demonstrated strong linkages between China Medical University and Shanghai Jiao Tong University, as well as between Harvard Medical School and the University of Florida (Figure 3B). In terms of publication volume, Chinese institutions were prominent, while U. S. institutions, such as the University of Florida, exhibited a high average of citations per paper. The top 10 institutions by publication count are summarized in Table 2, and their collaborative relationships are visualized in Figure 3B.

**Table 2:** Top 10 institutions by publication volume in oxidative stress-mediated brain-bone axis dysregulation research.

Rank	Organization	Country	Total link strength	Number of publications	Number of total citations	Number of average citations
1	Shanghai Jiao Tong University	China	49	19	414	21.7895
2	Cairo University	Egypt	18	14	206	14.7143
3	China Medical University	China	47	13	238	18.3077
4	Capital Medical University	China	15	9	156	17.3333
5	University of Florida	United States	10	8	745	93.125
6	National Research Centre	Egypt	15	7	114	16.2857
7	Fujian Medical University	China	9	6	101	16.8333
8	Harvard Medical School	United States	21	6	313	52.1667
9	Shandong University	China	5	6	98	16.3333
10	Sichuan University	China	14	6	206	34.3333

### 3.4 Key authors and collaborative networks

Analysis of the author collaboration network reveals the core researchers and their collaborative patterns in this field. As shown in Table 3, among the top 10 authors by publication volume, Yip, Hon-Kan and Sun, Cheuk-Kwan exhibit the strongest collaboration link strengths, with values of 42 and 39, respectively, indicating that they serve as the most active collaborative hubs within this research network. In terms of academic influence, Raizada, Mohan K. and Gendelman, Howard E. stand out most prominently, with their average citation counts per paper exceeding 125, highlighting the foundational role of their work. Furthermore, Zubcevic, Jasenka also demonstrates significant influence with a relatively high average citation count (91.25). Overall, the field has formed a collaboration network centered around authors with high link strength, while several key scholars with substantial influence have also emerged. The top 10 authors by publication volume are summarized in Table 3, and the author co-occurrence network is visualized in Figure 3C.

**Table 3:** Top 10 authors by publication volume in oxidative stress-mediated brain-bone axis dysregulation research.

Rank	Author	Total link strength	Number of publications	Number of total citations	Number of average citations
1	Grover, Paramjit	5	5	220	44
2	Yip, Hon-Kan	42	5	67	13.4
3	Gendelman, Howard E.	11	4	501	125.25
4	Raizada, Mohan K.	15	4	504	126
5	Sun, Cheuk-Kwan	39	4	67	16.75
6	Villarreal, Cristiane Flora	21	4	127	31.75
7	Wang, Jing	1	4	65	16.25
8	Zubcevic, Jasenka	15	4	365	91.25
9	Chai, Han-Tan	37	3	55	18.3333
10	Chen, Kuan-Hung	27	3	28	9.3333

### 3.5 Analysis of core journal and co-citation patterns

Analysis of journal distribution identified PLOS One, Proceedings of the National Academy of Sciences of the United States of America (PNAS), and the Journal of Biological Chemistry as prominent publishing platforms in this field. The journal co-citation network reflects the intellectual influence of multidisciplinary journals such as Nature and Science, alongside specialized periodicals including Free Radical Biology and Medicine and Brain Research (Figure 3D). The data indicated that highly cited articles from U.S. scholars were frequently found in Nature and Science, while institutions from China, notably Shanghai Jiao Tong University, maintained a high publication volume in PLOS ONE and International Journal of Molecular Sciences. The top 10 journals by publication volume are summarized in Table 4, and the journal co-occurrence map is visualized in Figure 3D.

**Table 4:** Top 10 journals by publication volume in oxidative stress-mediated brain-bone axis dysregulation research.

Rank	Journal	Total link strength	Number of total citations
1	Plos One	20,065	836
2	P Natl Acad Sci USA	21,346	764
3	J Biol Chem	19,190	753
4	J Pineal Res	16,240	619
5	J Neurosci	20,469	589
6	Nature	16,286	563
7	Int J Mol Sci	12,710	534
8	Free Radical Bio Med	10,529	451
9	Brain Res	11,398	433
10	Science	11,308	424

### 3.6 Analysis of keyword co-occurrence and research hotspots

**Figure 4:**
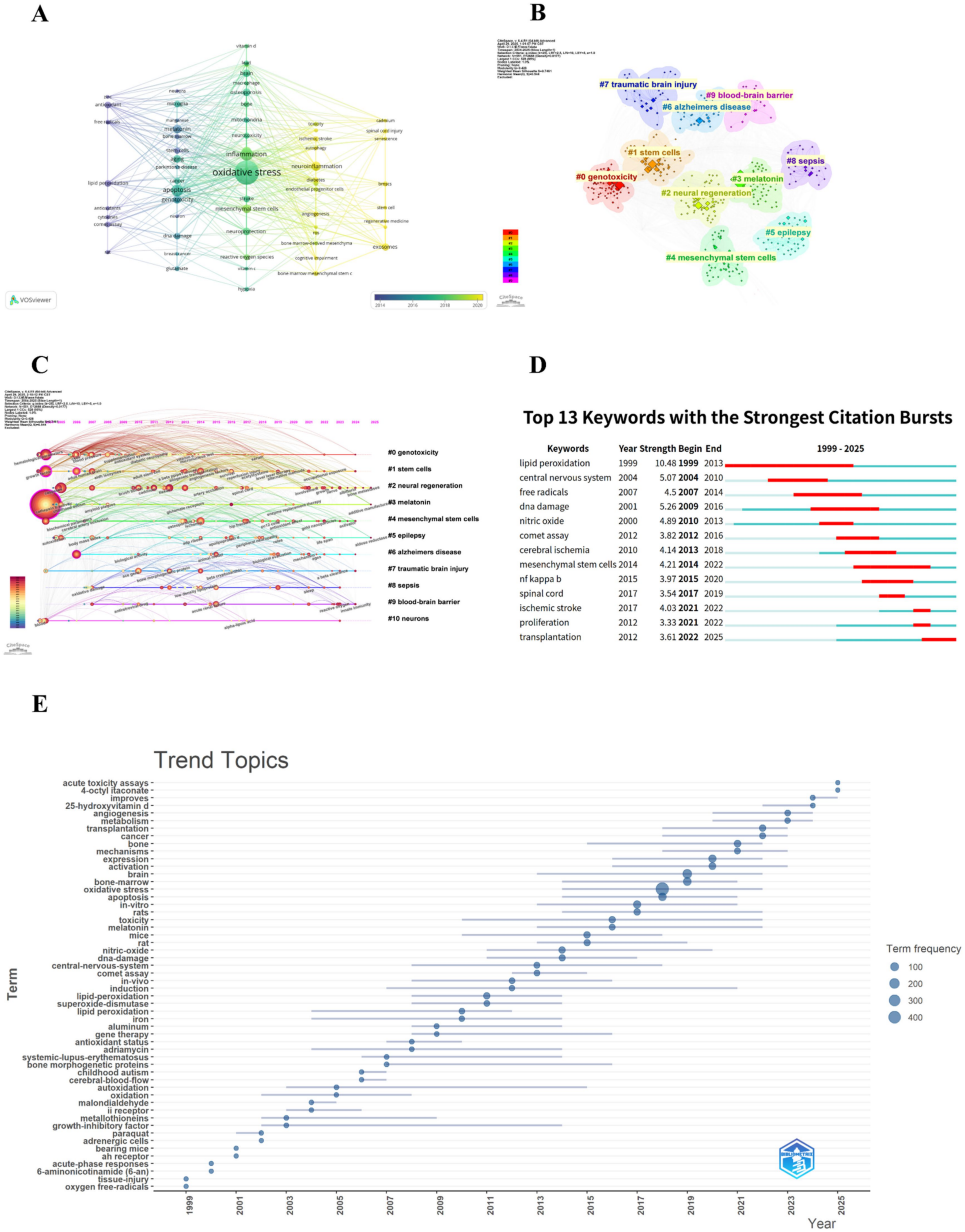
Multidimensional analysis map of keyword and time-series thematic trends in research on oxidative stress-mediated brain-bone axis dysregulation. **(A)** Keyword co-occurrence map: nodes represent keywords, their size reflects the frequency of co-occurrence, and connecting lines indicate associations between keywords. **(B)** Keyword clustering map: different colors represent distinct research theme clusters. **(C)** Keyword timeline map: keywords within the same cluster are arranged chronologically (1999–2025), with timeline length reflecting the duration of cluster research, node size indicating co-occurrence frequency, and connecting lines representing co-citation relationships. **(D)** Keyword burst detection map: displays the evolution of research frontiers, with red segments marking the time periods during which keywords emerged as research hotspots (Begin to End). Collectively, these reveal the research structure, thematic evolution, and frontier trends in the field. **(E)** Time series-thematic heat analysis map: illustrates the temporal trends of research theme popularity through the horizontal axis (year) and vertical axis (thematic terms), with the color bar on the right indicating the frequency of term occurrence.

Keyword co-occurrence analysis was conducted to identify core research themes and their structural relationships. The resulting map reveals that “oxidative stress” (frequency: 140, total link strength: 195; centrality: 0.52) occupies the most central position in the network, acting as a primary hub (Table 5). Significant associations were observed between “oxidative stress” and “inflammation,” which together form the structural backbone of the research network through 60 co-occurrence links. Additionally, “neuroinflammation” exhibited 26 co-occurrences and shared 20 links with “Alzheimer’s disease” (Figure 4A). Temporal analysis indicates that the focus of the keyword network shifted from “genotoxicity” (early peak around 2014) to “neuroinflammation” and related terms by 2020. The top 10 keywords by frequency and their corresponding centrality values are listed in Table 5, and the keyword co-occurrence network is visualized in Figure 4A.

**Table 5:** Top 10 keywords by publication volume in oxidative stress-mediated brain-bone axis dysregulation research.

Rank	Keywords	Centrality	Total link strength	Occurrences	Number of average annual publications	Number of average citations
1	oxidative stress	0.52	195	140	2017.6643	27.8214
2	apoptosis	0.04	68	39	2016.8718	26.6154
3	inflammation	0.05	66	43	2018.2326	42.4186
4	neuroinflammation	0.11	39	26	2019.8846	73.1923
5	alzheimer’s disease	0.13	37	26	2018.5385	32.2692
6	mesenchymal stem cells	0.03	33	22	2018.6818	34.0455
7	melatonin	0.07	33	23	2015.087	78
8	brain	0.15	31	18	2017.4444	33.5556
9	genotoxicity	0.03	31	21	2015.6667	31.9524
10	osteoporosis	0.16	30	18	2017.2222	51.3889

### 3.7 Identification of major research domains based on keyword clustering

Keyword clustering analysis categorized the research landscape into nine distinct modules (Clusters #0-#8), with a mean Silhouette value of 0.7672, indicating high cluster homogeneity (Table 6). Based on the clustering algorithms, the research network is organized around the following four primary thematic blocks: (1) Stem cell and neuroprotection (Clusters #0, #2, and #4): These clusters are characterized by keywords such as “mesenchymal stem cells,” “radiation-induced brain injury,” “neural regeneration,” and “oxidative stress regulation,” frequently involving the “SIRT1 signaling pathway” and “PC12 cell models.” (2) Neuroendocrine regulation (Clusters #3 and #7): Prominent terms include “melatonin,” “blood-brain barrier,” and “Alzheimer’s disease.” (3) Disease-specific oxidative profiles (Clusters #5 and #8): This module comprises terms related to “epilepsy” and “sepsis.” (4) Environmental factors (Cluster #1): This cluster represents research containing terms such as “occupational exposure,” “battery workers,” and “pteridine metabolism.” The detailed keyword clustering information is summarized in Table 6, and the visualization map is presented in Figure 4B.

**Table 6:** Clustering of keywords in research on the brain-bone axis disruption mediated by oxidative stress.

Clustering	Size	Silhouette	Clustering label
#0 genotoxicity	107	0.643	Mesenchymal stem cell; protective role; pc12 cell; ameliorate radiation-induced brain injury; sirt1 pathway
#1 stem cells	66	0.807	Pteridine metabolism; battery worker; radical generating agent; enone moiety; small multitarget molecule
#2 neural regeneration	60	0.638	Stem cell therapy; prefrontal cortex; modulating glutamatergic receptor; core symptom; low dose aluminium
#3 melatonin	59	0.71	Endothelial cell; vitamin c; marrow-derived mesenchymal stem cell; brain microvascular; blood–brain barrier-forming capacity
#4 mesenchymal stem cells	58	0.762	Thorium-induced neurobehavioural; post-traumatic dynamic change; neurochemical alteration; wistar albino rat; antibody titer
#5 epilepsy	55	0.751	Heterogeneous effect; catenin pathway; multiple site; bone-brain axis; craving reduction
#6 alzheimers disease	55	0.692	Protective effect; molecular mechanism; pc12 cell; adipose-derived mesenchymal stem cell; protective role
#7 traumatic brain injury	46	0.852	Common cancer chemotherapeutics; mammary cancer; health promotion; aging people; physical activities
#8 sepsis	29	0.842	Fluoride-induced hematological biochemical DNA damage; nutritional biochemistry perspective; health effect; murine model; macrophage delivery
#9 blood–brain barrier	20	0.975	Fat mobilization; lead-exposed mallard; fatty acid composition; alkaline phosphatase activity; rat brain homogenate

### 3.8 Temporal evolution of research themes based on keyword timeline analysis

The keyword timeline visualization (Figure 4C) illustrates the chronological progression and structural distribution of the research network. In the graphical representation, several interconnected clusters form the network architecture. The central region comprises clusters related to neural injury and neurological diseases, notably #6 Alzheimer’s disease, #5 epilepsy, #7 traumatic brain injury, #12 stroke, #15 schizophrenia, #14 autism, and #16 Down syndrome. Surrounding this assembly are thematic groups involving fundamental mechanisms, such as #0 genotoxicity, #9 blood–brain barrier, and #10 neurons; clusters representing intervention strategies, such as #1 stem cells, #4 mesenchymal stem cells, #3 melatonin, and #2 neural regeneration; and clusters reflecting systemic associations, such as #8 sepsis and #11 cardiovascular disease. Extensive linkages are observed between these modules. Based on the timeline view, the nodes exhibit a distinct distribution along the temporal axis. During the initial phase (2004–2010), nodes related to basic mechanisms, such as #0 genotoxicity, emerged earlier. In the subsequent period (2010–2020), nodes representing therapeutic application, specifically #1 stem cells and #4 mesenchymal stem cells, became prominent. In the most recent interval (2020–2025), nodes including #2 neural regeneration are positioned toward the right of the timeline, characterized by the emergence of newer labels and dense interconnections (Figure 4C).

### 3.9 Identification of research Frontiers based on keyword burst analysis

Keyword burst analysis was employed to identify keywords with significant fluctuations in citation frequency over time (Figure 4D). In the initial phase (1999–2013), bursts were observed for terms related to oxidative processes, including “lipid peroxidation” (1999–2013) and “free radical” (2007–2014), alongside co-occurring bursts of “DNA damage” (2009–2016), “nitric oxide” (2010–2013), and “central nervous system” (2004–2010). During the intermediate phase (2013–2020), the network was characterized by the emergence of “cerebral ischemia “(2013–2018),“spinal cord injury” (2017–2019), and the “NF-κB” signaling pathway (2015–2020), with “mesenchymal stem cells” (2014–2022) representing a sustained burst period. The most recent phase (2021–2025) is marked by burst terms such as “ischemic stroke” (2021–2022), “proliferation” (2021–2022), and “transplantation therapy” (2022–2025) (Figure 4D).

### 3.10 Temporal trends in research theme popularity based on heat analysis

To deepen the understanding of the historical context and developmental trajectory of research on brain-bone axis dysregulation mediated by oxidative stress, we generated a thematic trend map (Figure 4E) based on the core dataset of 717 publications constructed earlier. This map visualizes the annual frequency of high-occurrence thematic terms, thereby revealing the evolutionary path of overall hotspots in the field. In the early stage (approximately 1999–2005), research topics primarily focused on fundamental oxidative damage mechanisms, such as “oxygen free-radicals,” “tissue-injury,” “malondialdehyde,” and “autoxidation.” During the middle period (approximately 2006–2017), the research scope expanded significantly, delving into broader disease areas and tissue-specific investigations. The core term “oxidative stress” itself remained highly prominent. Disease-related research increased significantly, with topics like “cancer,” “systemic-lupus-erythematosus,” and “childhood autism” becoming hotspots. It is noteworthy that tissue-specific research themes such as “bone,” “bone-marrow,” and “central-nervous-system” began to emerge and remained active during this phase, while key mechanism-related themes like “apoptosis” and “DNA-damage” also maintained high prominence. In recent years (approximately 2018–2025), new trends have appeared at the research frontier. On one hand, studies targeting specific active substances and novel cell death modalities have become emerging hotspots, as evidenced by the significantly increased frequency of themes like “4-octyl itaconate,” “melatonin,” and “25-hydroxyvitamin d.” On the other hand, directions closely related to tissue repair and metabolism, such as “angiogenesis,” “metabolism,” and “transplantation,” have sustained continued interest. In summary, research hotspots in the field of oxidative stress-mediated brain-bone axis dysregulation have undergone an evolutionary journey: from early exploration of fundamental mechanisms, to mid-period association with a wide range of diseases and specific organs, and recently to a focus on frontiers involving novel cell death modalities and immunometabolic regulation.

### 3.11 Analysis of highly co-cited foundational publications

Document co-citation analysis identified 10 foundational publications with high co-citation frequency (Table 7). The publication years of these highly cited works span from 1951 to 2009. Based on document type, the collection comprises methodological papers (*n* = 5), stem cell research articles (*n* = 3), and review articles (*n* = 2). Temporal analysis shows that methodological studies were published primarily between the 1950s and 1990s, while stem cell research and theoretical reviews are concentrated in the period after 2000. Content analysis reveals that co-cited methodological literature predominantly addresses key analytical protocols, including protein quantification, lipid peroxidation, and DNA damage detection. The core themes of the stem cell publications involve the multilineage differentiation potential of mesenchymal stem cells and their standardized definition, while the review articles discuss the association between oxidative stress and neurodegenerative diseases. Detailed information on these foundational works is summarized in Table 7.

**Table 7:** The top 10 co-cited papers in the field of oxidative stress-mediated brain-bone axis disorders research.

Rank	First author	Centrality	Year	Journal	Article type	Key contributions
1	Lowry et al., (1951)	0.13	1951	J Biol Chem	Methodological development	Developed the Folin-phenol reagent method for protein quantification, utilizing tyrosine/tryptophan reduction of phosphotungstic-phosphomolybdic acid to form blue complexes. Established as the standard biochemical method for protein concentration measurement.
2	Ohkawa et al. (1979)	0.05	1979	Anal Biochem	Methodological development	Created the thiobarbituric acid (TBA) reaction assay for lipid peroxidation products (e.g., MDA), quantifying red chromogen formation at 532 nm. Became a classical tool for oxidative stress assessment.
3	Tice et al. (2000)	0.04	2000	Environ Mol Mutagen	Guideline/consensus statement	Established international guidelines for alkaline single-cell gel electrophoresis (Comet assay), standardizing critical steps (lysis, alkali treatment, electrophoresis) for DNA damage detection.
4	Dominici et al. (2006)	0.04	2006	Cytotherapy	Guideline/consensus statement	Proposed minimal criteria for defining human mesenchymal stem cells (MSCs): plastic-adherence property, specific surface markers (CD105+/CD73+/CD90+; CD45-/CD34-/HLA-DR-), and trilineage differentiation potential. Resolved comparability issues in MSC research.
5	Livak and Schmittgen (2001)	0.04	2001	Methods	Methodological development	Systematized the 2^−ΔΔCt^ method for relative quantification in real-time PCR (qPCR), calculating expression fold-changes using dual ΔCt differences between target and reference genes. Became the standard framework for gene expression analysis.
6	Pittenger et al. (1999)	0.02	1999	Science	Experimental research	First demonstration of multilineage differentiation potential (osteogenic, chondrogenic, adipogenic) in adult bone marrow MSCs. Established surface marker identification criteria, challenging conventional paradigms of adult stem cell unipotency and laying theoretical foundations for regenerative medicine.
7	Singh et al. (1988)	0.02	1988	Exp Cell Res	Methodological development	Pioneered single-cell gel electrophoresis (comet assay), enabling quantitative assessment of DNA damage in individual cells through alkaline-induced DNA fragmentation patterns (“comet tails”). Became a simple yet powerful tool in genetic toxicology and disease pathology evaluation.
8	Bradford (1976)	0.04	1976	Anal Biochem	Methodological development	Introduced the Coomassie Brilliant Blue G-250 dye-binding method for rapid protein quantification. Detects microgram-level protein concentrations within 5 min through absorbance shifts, replacing the Lowry method as the routine laboratory protocol due to its simplicity.
9	Caplan and Dennis (2006)	0.04	2006	J Cell Biochem	Review	Proposed the “trophic microenvironment” theory: MSCs mediate tissue repair and immunomodulation through paracrine secretion of growth factors, cytokines, and extracellular vesicles. Redefined theoretical frameworks for stem cell therapies.
10	Uttara et al., (2009)	0.02	2009	Curr Neuropharmacol	Review	Systematically examined pathological links between oxidative stress and neurodegenerative diseases. Proposed an “upstream prevention-downstream clearance” antioxidant strategy, emphasizing tiered interventions for guiding neuroprotective therapies.

## 4 Discussion

### 4.1 Thematic evolution: from molecular mechanisms to systems integration

The trends identified in this study reflect a significant transition in how oxidative stress-mediated brain-bone axis dysregulation is investigated. Initially, scientific inquiry was predominantly confined to the independent roles of oxidative stress within isolated neural or skeletal systems, focusing on ROS-induced damage to cell membranes and DNA. However, current thematic clustering (Section 3.7) demonstrates an expansion of research scope toward an integrated understanding of bidirectional interaction mechanisms (Li et al., 2024a,b). This research framework emphasizes that the central nervous system regulates bone remodeling via neurotransmitters, while bone-derived factors—notably osteocalcin and fibroblast growth factor (FGF)—traverse the blood–brain barrier to modulate neurogenesis and cognitive function (Parthasarathy et al., 2023; Stephan and Sleiman, 2021). Such bidirectional crosstalk establishes oxidative stress not merely as a byproduct of localized damage, but as a “common pathological basis” that links neuro-skeletal comorbidities. For instance, evidence suggests that chronic stress-induced elevation of brain ROS activates the HPA axis, triggering glucocorticoid secretion that consequently suppresses osteoblast activity (Prevatto et al., 2017); conversely, the systemic influx of bone-derived inflammatory factors can exacerbate neuroinflammation and oxidative damage (Pan et al., 2025), creating a self-reinforcing cycle of axis dysregulation.

### 4.2 Core research dimensions: mechanisms, associations, interventions, and methodologies

Based on keyword co-occurrence and burst-term analysis, the current research landscape can be categorized into four primary dimensions: molecular mechanisms, disease associations, intervention strategies, and technical methodologies. At the molecular mechanism level, ROS signal transduction, the Nrf2/ARE pathway, and mitochondrial dysfunction form the core research clusters. Extensive studies demonstrated that ROS not only directly damage neurons and bone cells by activating signaling pathways such as NF-κB and MAPK, but also disrupt the RANKL/OPG system, thereby influencing the process of bone remodeling (Ma et al., 2022; Wang et al., 2023; Yan et al., 2023). Notably, the dual regulatory role of the Nrf2-mediated antioxidant defense system has become a central focus over the past 5 years (Che et al., 2023; Zgorzynska et al., 2021). Secondly, regarding disease associations, the comorbidity mechanism of Alzheimer’s disease and osteoporosis, abnormal bone metabolism in depression, and fracture risk in Parkinson’s disease constitute the three major clinical hotspots (Ahn et al., 2025; Skowronska-Jozwiak et al., 2020; Wanionok et al., 2024). This close association is further supported by the bidirectional effects of brain-derived neurotrophic factor and bone-derived osteocalcin on cognitive function and skeletal homeostasis (Camerino et al., 2016).

Furthermore, in terms of intervention strategies, natural antioxidants (such as resveratrol and curcumin), exercise and gut microbiota have emerged as prominent treatment modalities. Specifically, resveratrol exerts dual benefits by concurrently activating SIRT1 and Nrf2 pathways (Peng et al., 2024; Zhang et al., 2022). The role of the microbiota–gut–brain-bone axis is increasingly prioritized, where the microbiota modulates brain–bone function through the synthesis of neurotransmitters and metabolites (Ding et al., 2020; Parker et al., 2024; Sun et al., 2021). Short-chain fatty acids can inhibit osteoclast differentiation and alleviate neuroinflammation by activating the aryl hydrocarbon receptor in microglia; simultaneously, pro-inflammatory factors driven by dysbiosis can affect bone health through humoral or cellular immune pathways, promoting osteoclast activation. In contrast, probiotics help maintain skeletal integrity by balancing of Treg/Th17 cells (Grüner et al., 2023; Lee et al., 2018; Sashide et al., 2025). Additionally, bones tissue communicates bidirectionally with the intestinal nerve plexus and the brain through cytokines (such as neuropeptide Y) to form an upward pathway within the bone (Li et al., 2021; Tang and Cao, 2021; Zhang et al., 2023a,b). Finally, the dimension of technical methodologies, including single-cell transcriptomics and organoid co-culture, continues to reshape the field by providing high-resolution characterization of these complex inter-system communications.

### 4.3 Methodological innovation and research Frontiers

The trajectory of brain-bone axis research is increasingly defined by the integration of high-resolution biotechnologies and systemic modeling. Methodologically, the convergence of single-cell transcriptomics, organoid co-culture systems, and multi-omics analysis has established a new technical frontier. Notably, the application of single-cell RNA sequencing has proven instrumental in deconvoluting the cellular heterogeneity within the blood–brain barrier and bone microenvironment, specifically regarding how diverse cell populations respond to oxidative stress (Liao et al., 2025; Yang et al., 2022). Furthermore, the incorporation of spatial transcriptomics has enabled the mapping of ROS distribution at specific anatomical interfaces, providing a structural context to redox signaling that was previously unattainable.

This technological evolution is driving a fundamental transition from single-organ investigations toward integrated multi-system frameworks, such as the “brain–bone–gut” and “brain–bone–immune” axes. Current trends prioritize the role of gut microbiota metabolites in modulating redox balance across these systems (Li et al., 2024a,b). At the interface of these disciplines, the maturation of spatiotemporal multi-omics—combining single-cell resolution with spatial metabolomics—allows for the precise characterization of ROS dynamics within the neuro-skeletal niche (Marshe et al., 2025). Beyond biological assays, the explosive growth of artificial intelligence (AI)-assisted drug screening is accelerating the identification of potent antioxidants (Wu et al., 2024). These advancements, complemented by organoid-on-a-chip technologies, are successfully simulating brain-bone axis functions for high-throughput screening, thereby narrowing the gap between basic mechanistic discovery and preclinical evaluation.

### 4.4 Clinical translation, global research trends, and future directions

In the realm of clinical translation, the principles of precision medicine are fundamentally reorienting the therapeutic development of the brain-bone axis. Current research focus has transitioned toward individualized antioxidant strategies, characterized by a shift from single-biomarker screening to integrated diagnostic profiles, and from broad-spectrum supplementation to targeted drug delivery systems. Simultaneously, efficacy evaluation has expanded from traditional clinical symptoms to comprehensive multi-omics endpoints. Notably, the past 3 years have seen a significant surge in clinical interest regarding non-pharmacological interventions, such as photobiomodulation and transcranial electrical stimulation, aimed at modulating neuro-skeletal function (Cao et al., 2026; Liu et al., 2026). The global distribution of research highlights a high degree of geographic concentration, with China and the United States maintaining the most prominent roles in collaborative networks. However, a distinct divergence in research priorities is evident: Chinese teams focus heavily on the neuro-osteological protective mechanisms of traditional medicine-derived active ingredients, whereas American teams exhibit a preference for the development of novel synthetic antioxidants and advanced disease-modeling technologies.

Looking forward, this field is poised to advance along several critical frontiers over the next decade. Basic research will increasingly prioritize microenvironmental heterogeneity and intercellular communication, while technological development is expected to center on live dynamic imaging and digital twin models. From a clinical perspective, exploring the synergistic effects of antioxidant treatments with existing therapies remains a priority. Furthermore, as epigenetic research deepens, the role of DNA methylation-mediated oxidative stress memory in the long-term regulation of the brain-bone axis represents a promising breakthrough in understanding chronic neuro-skeletal dysregulation.

### 4.5 Study limitations

Several limitations inherent in this bibliometric analysis must be acknowledged. First, although this study integrated data from the WoSCC, Scopus, and PubMed databases, it may not fully capture non-English publications or regionally specialized studies absent from these platforms, potentially introducing a degree of geographic or linguistic bias. Second, the lack of standardized protocols for quantifying oxidative stress across different laboratory settings limits the direct comparability of findings, as methodological variations in biomarker assays can influence results. Furthermore, the current reliance on acute injury models to simulate oxidative stress-mediated pathology fails to fully replicate the progressive and chronic nature of human neuro-skeletal conditions. These constraints underscore the necessity for future research to establish standardized assessment frameworks and prioritize the development of physiologically relevant chronic models to better translate basic mechanistic insights into clinical applications.

## 5 Conclusion

Using a multi-database and multi-software bibliometric approach, this study systematically mapped the intellectual structure, developmental trajectory, and thematic evolution of research on oxidative stress-mediated brain-bone axis dysregulation. The findings indicate that oxidative stress is increasingly conceptualized not simply as a localized biochemical disturbance, but as a central pathological link connecting neurodegenerative disorders and metabolic bone disease.

By integrating co-occurrence mapping, clustering analysis, and burst-term detection, this study demonstrates a clear shift in research focus from isolated, organ-centered investigations toward multi-system frameworks, most notably the emergence of the “brain–bone–gut” axis as a prominent research theme. This evolution reflects a broader recognition that the neuro-skeletal interface functions as a dynamic and systemically responsive redox network rather than as two independent physiological compartments.

This thematic maturation is accompanied by a significant shift toward clinical translation, as investigations have increasingly progressed from the fundamental characterization of ROS signaling pathways, such as Nrf2/ARE, to the evaluation of targeted interventions and non-pharmacological therapies. Simultaneously, methodological refinements are evident, where traditional bulk biochemical assays are being complemented by high-resolution spatiotemporal characterization, driven by the integration of single-cell sequencing and organoid technologies. These transitions reflect an expanding research scope aimed at resolving the cellular heterogeneity and niche-specific signaling inherent in neuro-skeletal crosstalk. Accessing these high-resolution data is essential for identifying specific molecular markers of brain–bone communication across different stages of aging.

Despite these advancements, the analysis identifies notable research gaps, particularly the lack of standardized protocols for assessing oxidative stress and an over-reliance on acute injury models that may not fully reflect the progressive nature of chronic pathology. Future research efforts should therefore prioritize the integration of cross-scale multi-omics data and the development of physiologically relevant chronic models to align mechanistic discovery more closely with clinical phenotypes. Furthermore, the potential role of epigenetic “redox memory” in long-term brain-bone axis regulation remains a promising area for exploration. Ultimately, this knowledge mapping provides a systematic reference for researchers to better understand the complex interdisciplinary landscape, reinforcing the brain-bone axis as a vital frontier for precision intervention in age-related comorbidities.

## Data Availability

The datasets presented in this study can be found in online repositories. The names of the repository/repositories and accession number(s) can be found at: https://doi.org/10.5281/zenodo.20340267.
